# Which Physical Quantity Deserves the Name “Quantity of Heat”?

**DOI:** 10.3390/e23081078

**Published:** 2021-08-19

**Authors:** Friedrich Herrmann, Michael Pohlig

**Affiliations:** Karlsruhe Institute of Technology, 76128 Karlsruhe, Germany; pohlig@kit.edu

**Keywords:** heat, balance equation for heat, entropy as heat, enthalpy, thermal energy, caloric

## Abstract

“What is heat?” was the title of a 1954 article by Freeman J. Dyson, published in Scientific American. Apparently, it was appropriate to ask this question at that time. The answer is given in the very first sentence of the article: heat is disordered energy. We will ask the same question again, but with a different expectation for its answer. Let us imagine that all the thermodynamic knowledge is already available: both the theory of phenomenological thermodynamics and that of statistical thermodynamics, including quantum statistics, but that the term “heat” has not yet been attributed to any of the variables of the theory. With the question “What is heat?” we now mean: which of the physical quantities deserves this name? There are several candidates: the quantities Q, H, $E_{\mathrm{therm}}$ and S. We can then formulate a desideratum, or a profile: What properties should such a measure of the quantity or amount of heat ideally have? Then, we evaluate all the candidates for their suitability. It turns out that the winner is the quantity S, which we know by the name of entropy. In the second part of the paper, we examine why entropy has not succeeded in establishing itself as a measure for the amount of heat, and we show that there is a real chance today to make up for what was missed.

## 1. Introduction

Today we have a comprehensive knowledge about how states and processes which we consider to belong to thermodynamics can be described macroscopically as well as microscopically. The development that led to this state of the knowledge proceeded parallel to that of the other classical subfields of physics and reached a certain completion with the work of Gibbs and Boltzmann at the end of the 19th century.

A question that has accompanied this development of thermodynamics from the beginning was: what is heat?

This question assumes that heat is something that exists in the real world, and that it is the task of physics to find out what its nature is, and what properties it has. The given answers are well-known: heat is a substance, heat is the movement of the molecules, heat is a form of energy, heat is disordered energy. 

However, a more modern, enlightened and perhaps somewhat disappointing answer to the question would be: heat is the name given to a physical quantity, i.e., a variable in a well-working theory.

In the following, we are only concerned with whether the assignment of this name to the quantity Q was a lucky move. Is there a physical quantity to which the designation heat fits better than to Q? Which quantity deserves the name?

Let us pose the question more precisely. In the following, by heat we do not mean “being hot”, which would be measured by the quantity temperature; we mean a physical quantity that could also be called the quantity of heat or amount of heat.

So, once again, which physical quantity deserves this name?

Let us imagine that thermodynamics exists as a theory in its modern form. However, the physical quantities appearing in the theory are only denoted by symbols; as yet they have no names. We know the measuring procedures for all of them, but they are not called pressure, temperature, internal energy, etc., but p, T, U, etc. We now want to give suitable names to these quantities. In particular, we are concerned with whether one of the quantities deserves the name heat or amount of heat, and if so, which one.

We can think of several claimants for the term “heat”. Of course, among them is the quantity Q (which received the term after all), but also the quantities H and S (today called “enthalpy” and “entropy”). Another candidate is a quantity which does not have a symbol of its own, and which is called “thermal energy”. Let us refer to it as $E_{\mathrm{therm}}$. All of these quantities have in common that in some context one actually deals with them as with measures of an amount of heat.

To find the best candidate for the name heat, we must first explain what we expect from such a quantity. In fact, it is not difficult to formulate a set of criteria, see Section 2. We will create a “profile” for the amount of heat.

In Section 3, we examine which of the candidates best meets the criteria of our profile and it turns out that there is a clear winner: entropy.

In Section 4, we briefly describe the historical evolution of the quantity which in the end was named “entropy”, one can also say: its tragic fate. Generally speaking, it had three chances to establish itself as a measure of the amount of heat. At least the first and the second opportunities were missed.

One might think that it is not very important which name is given to a physical quantity; it is only important that it has a name at all, so that one can refer to it. The experience of many teachers and lecturers shows that this is not true. A well-chosen name can be an important aid to learning, and a poorly chosen one a hindrance to understanding. We will show in Section 5 the consequences that the choice of the term “heat” and its assignment to a physical quantity has for teaching.

## 2. Profile of a Physical Quantity *Amount of Heat*

One might think that such a profile contains arbitrariness and that one cannot answer the question of which quantity deserves the term in this way. In fact, it is not at all difficult to formulate some maximum requirements, even if one may fear that there is no physical quantity which satisfies all of them. 

The expectations or requirements for a quantity *amount of heat*, which we formulate, are those of the scientist. However, we will see that they could be formulated quite similarly by a layperson. Actually, the non-professionals have an advantage: they are not biased. The bias of the person educated in physics could be, for example, that they take it for granted that heat must be an energetic quantity.

Here is our profile:

1. Heat is contained in a body. It is distributed within the body. Two bodies, one of which contains 3 units of heat and the other 2 units, together contain 5 units;
2. If heat is added to a body, its temperature usually rises. It may happen that the temperature does not rise despite the supply of heat, for example when water is boiling. However, now the water evaporates. Steam must therefore contain more heat than liquid water of the same temperature (scalding with steam is known to be worse than with boiling water). One can also increase the temperature of a gas by compressing it, and thereby compressing the heat;
3. Heat can pass or flow from one body to another. If there is a suitable connection between two bodies, heat passes or flows “by itself” from the warmer to the colder one;
4. Heat is generated in a flame and in many other chemical reactions, as well as by friction and by electric currents; it is not taken away from another system in these processes, but is generated anew.

We might come up with more demands or wishes for our profile, but those compiled here are already sufficient to clearly assess the candidates of our list. 

Some of these requirements (1, 3 and 4) can be summarized in a mathematical expression. Let us call our still hypothetical heat measure X. We can use it to formulate an equation that makes a statement about a given region of space; the space, for example, that is occupied by a body:

(1) $$\frac{dX}{dt}+I_{X}=\sum_{X}$$

where dX/dt is the time rate of change of X in our region of space; $I_{X}$ is the flow through the surface of the region; and $\sum_{X}$ is the production rate, again within our region. The equation tells us that the heat content of the region can change in two ways: by a flow through the surface, or by production inside the region. It expresses the fact that for heat a balance can be established. One can formulate this fact even more elegantly, namely locally via:

(2) $$\frac{\partial \rho_{X}}{\partial t}+div\;j_{X}=\sigma_{X}$$

where $\rho_{X}$ is the density, $j_{X}$ is the current density and $\sigma_{X}$ is the “production density” of X.

Let us call this equation the local balance equation of X. In another context, it would also be called a continuity equation.

Thus, we would like a physical quantity “heat” to meet the expectations formulated above as far as possible. Let us use them to assess the suitability of our candidates.

## 3. The Candidates

We have already mentioned the candidates. They are:

1. Q
2. H
3. $E_{\mathrm{therm}}$
4. S

They bear the names heat, enthalpy, thermal energy, and entropy.

The fact that they are used as a measure of heat can be seen from the way the quantities are handled. We will return to this later. In the first three cases however, it can also be recognized by the names given to them. The names for Q and $E_{\mathrm{therm}}$ speak for themselves, and the term “enthalpy” is clear enough if one considers its Greek root: ἐν, “in,” and θάλπειν, “to heat”.

Only entropy has a name which does not express its relation to a system’s state of being cold or hot. According to Clausius, it is a measure for a transformation. Therefore, at first he called the quantity “transformation” (Verwandlung) or, to emphasize the substance-like nature of the quantity, “transformation content” (Verwandlungsinhalt). Only later did he suggest the name entropy [1]:

“If one looks for an appropriate name for *S*, then one could say the quantity S to be the transformation content of the body, similarly as the quantity U is said to be the heat and work content of the body. However, since I think it is better to choose the names of quantities, which are important in science, from the old languages, so that they can be applied unchanged in all the new languages, I propose to call the quantity S according to the Greek word ητροπη, the transformation, the entropy of the body.”

We now review the candidates, one by one, and examine the extent to which they meet the criteria of our profile.

First, we recall their definitions as typically found in textbooks. We take them from various sources but we do not specify these sources, because it is not our intention to criticize specific authors. We assume that the reader will realize that the definitions are typical and commonly used. We look at how they are used in physics as well as in chemistry to describe processes involving a “heat balance”. 

We then examine which of the criteria of our profile are met by the variable under consideration and address the problems associated with not meeting one or more of the criteria. 

### 3.1. The Quantity Q

#### 3.1.1. Definition and Use

In textbooks, one can find statements such as the following:

“The physical quantity heat describes a part of the energy absorbed or supplied by a system. The remaining part is work.”

“Heat is energy in transfer to or from a system A to a system B by a mechanism other than work or transfer of matter.”

“The change of the energy due to the energy transport as a result of a purely thermal interaction is called heat.”

Usually, the quantity Q is simply called “heat”, sometimes “heat energy” and sometimes “amount of heat.” 

We encounter it in the first law of thermodynamics, which can be expressed as:

(3) ΔU=Q−W

or in order to emphasize the fact that Q and W are not variables in the usual sense:

(4) dU= δQ− δW

A comparison with Gibbs’s fundamental equation:

(5) $$dU=TdS-pdV+\Sigma \mu_{i}\;dn_{i}$$

shows that we can also write:

(6) δQ=TdS .

Thus, if the entropy has already been introduced, heat can also be defined by Equation (6). The equation shows that Q is used to describe processes in which energy is transferred together with entropy, and in this case the transferred energy is called heat.

Q is not a variable in the sense of mathematics. It is a so-called differential form. Besides Q physics knows only one other construction of this kind: the work W. This peculiarity is expressed by saying that Q and W are “process functions”. In fact, one could create several other expressions of this kind and give each of them a name of its own.

#### 3.1.2. Which Criteria of the Profile Are Met

Criterion 1 is not satisfied, because Q is not a state variable. There is no possibility of or logic in assigning a value of Q to a given system in a given state. Therefore, one cannot say Q is contained in a body. It also follows that one cannot formulate a balance equation for Q. 

Moreover, criterion 2 is not met. If Q does not have a value for a given system, one cannot establish a relationship between Q and the temperature, nor is it possible to say that water vapor contains more Q than liquid water of the same temperature.

Strictly speaking, the quantity does not fulfill criterion 3 either. Although one often says in the context of physics that a system releases or receives heat (Q), one must bear in mind that here the terms “receive” and “release” are used in a form different from colloquial language.

If we say in common language that a system A releases something, let us call it X, and B receives it, it is taken for granted that X was contained in A before, and is contained in B afterwards.

For criterion 4 the same is valid as for criterion 3. It is common in physics to speak of heat production, but this way of speaking is again contrary to the logic of the colloquial language. If one says, something, let us call it X again, is produced, then, according to common understanding, this X is present somewhere afterwards. This is not true however, if one uses Q for X, because Q is not present anywhere. 

The preceding discussion shows that the “quantity” Q is cumbersome. The colloquial language, on which we also depend in physics, and which proves to be especially suitable when talking about extensive quantities, is not only unhelpful, it also leads to a wrong understanding of the quantity Q. 

### 3.2. The Quantity H

#### 3.2.1. Definition and Use

Enthalpy is defined as:

(7) H=U+pV

It is widely used in chemistry. The standard enthalpies of formation of many substances are listed in tables. For isobaric processes, the following applies to the changes of enthalpy:

(8) ΔH=Q

The relationship has the character of an energetic balance equation: on the right side the energy supplied to the system under consideration, and on the left side the change of an extensive quantity, measured in joules. It seems that H and Q together allow the establishment of a heat balance.

Here are some definitions from textbooks that mention its importance as a measure of heat:

“When a process occurs at constant pressure, the heat released or absorbed is equal to the change in enthalpy. Enthalpy (H) is the sum of the internal energy (U) and the product of pressure and volume (pV) given by the equation:

$$H=U+pV\;.{}^{\prime \prime }$$

“Enthalpy H is a thermodynamic state variable. It is a designation for the amount of heat given off or absorbed by a reaction. It is measured in kJ (kilojoules). One cannot measure the enthalpy of a state, but only the difference between two states.”

#### 3.2.2. Which Criteria of the Profile Are Met

The values of the enthalpy refer to a region of space. Therefore, criterion 1 is fulfilled. Criterion 2 seems to be fulfilled if we restrict ourselves to processes at constant pressure. That this is not true in general becomes particularly apparent when we consider an extreme case: a rigid, non-deformable body. If we increase the pressure in the body (say, by placing it in a liquid bath and increasing the pressure of the liquid), its enthalpy increases by any desired amount, without any energy or other extensive quantity being exchanged. So our criterion 2 is strongly violated. Criterion 3 can be regarded as fulfilled. Things look bad again for criterion 4. We consider a flow through a pipe and make sure that no energy is exchanged through the wall of the pipe. For such a flow, the enthalpy transported through every cross-section of the pipe is equal. Now, if there is a resistance to the flow anywhere in the pipe, a friction process takes place without changing the enthalpy flow, contradicting our criterion 4, which requires that heat is generated when there is friction.

### 3.3. The Quantity $E_{\mathrm{therm}}$

#### 3.3.1. Definition and Use

The use of the expression thermal energy is not consensual. Predominantly the word is used in the following sense: it is a part of the internal energy (occasionally, however, the quantity Q is also called thermal energy) [2,3]).

What part it is, is explained somehow like this:

“The potential and the kinetic energy of the particles together are also called thermal energy.”

“The total energy of a thermodynamic system, which consists of thermal energy (potential and kinetic energy of the particles), of chemical energy and nuclear energy, is the internal energy U.”

“Thermal energy refers to the energy contained within a system that is responsible for its temperature. Heat is the flow of thermal energy.”

#### 3.3.2. Which Criteria of the Profile Are Met

The question of which criteria of our profile are met, has a very short answer: none at all. It is a matter of course that one can specify a value for a physical quantity, which one obtains from a measurement or a calculation, but this is not the case with thermal energy. 

Introducing a quantity “thermal energy” in this way is wishful thinking. Apparently, it is assumed that the total energy E can be decomposed into summands, each of which depends on a different independent variable, for instance [4]:

(9) $$E\;\left(S,\;V,\;n,\;\ldots \right)=E_{0}+E_{1}\left(S\right)+E_{2}\left(V\right)+E_{1}\left(n\right)+\cdots$$

If this were possible, it would be logical to give names to the various terms and to interpret them accordingly, e.g., $E_{1}\left(S\right)$ would be the thermal and $E_{3}\left(n\right)$ the chemical part; $E_{2}\left(V\right)$ could be called compressional energy. In fact, such a decomposition is possible only in some special cases and only as an approximation, for example, when the total energy of a body is decomposed into rest energy and kinetic energy:

(10) $$E\;\left(p,\;S,\;\ldots \right)=E_{0}\left(S\right)+\frac{p^{2}}{2m}$$

However, this decomposition only works so long as the velocity of the body is small compared to the terminal velocity *c*, the speed of light.

Sometimes one does not notice that such a decomposition of the energy is not possible. Consider, for example, an ideal gas whose particles have no degrees of freedom other than translation: no oscillations, no electronic excitations, and no chemical or other reactions take place. 

Thus, the introduction of such a concept of “thermal energy” seems to be a desperate but failed attempt to define an energetic measure of the heat content of a system that has a certain vividness.

### 3.4. The Quantity S

#### 3.4.1. Definition and Use

As is well known, according to Clausius [5] *S* is introduced as:

(11) $$S\left(2\right)-S\left(1\right)=\int_{1,\;\mathrm{rev}}^{2}\frac{\delta Q}{T}$$

Since it is difficult to get a clear idea of the quantity on the basis of this definition, one usually switches to the microscopic statistical interpretation. Such an interpretation is correct, but unsatisfactory in so far as the other thermodynamic quantities are not explained statistically within the framework of phenomenological thermodynamics either, even though this is quite possible: for example, temperature and chemical potential as parameters in the Boltzmann distribution. The elusiveness resulting from its definition (11) has earned entropy the reputation of being one of the most difficult quantities in physics. However, it gradually became clear that one can form a very vivid idea of entropy. It can be considered as a measure of the quantity of heat, the subject explored in this article. We address how this occurred in Section 4. First, we submit it to the same procedure as our other candidates and check which of our criteria for a measure of heat it satisfies. 

#### 3.4.2. Which Criteria of the Profile Are Met

It may come as a surprise that it is precisely the entropy which fulfills all of our criteria; it is an extensive quantity and it is a state variable. A hot body contains more entropy than a cold one; water vapor contains more entropy than liquid water of the same temperature; it flows through a heat conductor from hot to cold; and it is generated in “frictional processes” of all kinds, including free-running chemical reactions. Due to this, one can formulate a local continuity equation. Finally, if one compresses a gas, taking care that no entropy escapes, the temperature of the gas will rise.

## 4. How It Came about That the Simple Meaning of Entropy Was Not Recognized—A Drama in Three Acts

After the preceding considerations we can conclude that something must have gone wrong in the development of thermodynamics. The fact that the quantity which we call entropy today had the simple properties of a heat measure, was noticed and emphasized several times in the course of the history of thermodynamics. This development of the concept of heat and entropy is documented in an abundant body of literature [6,7,8,9,10,11,12]. 

In order to understand these historical developments, we must bear in mind that the common view which prevailed was that heat was something provided for us by nature, and that it only had to be discovered. One was not sufficiently aware that physical quantities were invented or defined by humans, and that one could assign the name of a quantity the way one wished to, i.e., the most convenient.

Here, we only comment on those stages that are most important in our context. We will discover that heat had three chances to escape its unfortunate fate, which can be described as a drama in three acts.

### 4.1. Act One: The Old Heat and Its Decline

#### 4.1.1. Joseph Black—The First Measure for the Amount of Heat

In his lectures on the elements of chemistry published in 1807, Joseph Black distinguishes between a measure of an intensity and a measure of the quantity or amount to describe thermal processes [13] (p. 78):

“If, for example, we have one pound of water in one vessel, and two pounds in another, and these two quantities of water are equally hot, as examined by the thermometer, it is evident, that the two pounds must contain twice the quantity of heat that is contained in one pound. Undoubtedly, we can suppose that a cubical inch of iron may contain more heat than a cubical inch of wood, heated to the same degree; and we cannot avoid being convinced of this by daily experience.”

Black introduces a quantity of heat that meets all the requirements of an operational definition. It is also clear for him that heat can be produced [13] (p. 32):

“The heat producible by the strong friction of solid bodies, occurs often in some parts of heavy machinery, when proper care is not taken to diminish that friction as much as possible…”

And of course, Black’s heat is a state variable.

Thus, there are good reasons to identify in retrospect his quantity of heat with entropy, or at least to consider it as a precursor of entropy [14].

#### 4.1.2. Sadi Carnot—Heat Engine and Water Wheel

Shortly thereafter, in 1824, Carnot’s *Réflexions sur la puissance motrice du feu et sur les machines propres à développer cette puissance* [15] appeared. Carnot’s considerations start from Black’s concept of heat. 

For Carnot, Black’s heat concept obviously belongs to the accepted tools of the theory of heat. The question of what is the nature of heat was not important to him. The two terms he uses, namely “Chaleur” and “Calorique”, do not express any essential difference for him, as one can see in the following statement [15] (p. 15):

“We find it useless to explain here what is the quantity of caloric or quantity of heat (for we use the two expressions indifferently), nor to describe how these quantities are measured by the calorimeter. Nor will we explain what latent heat, degree of temperature, specific heat, etc. is. The reader must become familiar with these expressions by studying elementary treatises on physics or chemistry.”

His most important insight is that there is an analogy between the functioning of a heat engine (machine à feu) and that of a water wheel [15] (p. 28):

“According to the notions established so far, the motive power of heat can fairly be compared to that of a waterfall: both have a maximum that cannot be exceeded, whatever on the one hand the machine used to receive the action of water, and whatever on the other hand is the substance employed to receive the action of heat.

The motive power of a waterfall depends on its height and the amount of liquid; the motive power of heat also depends on the quantity of caloric employed, and what might be called, what we shall call the height of its fall, that is to say, the difference in temperature of the bodies between which is the exchange of caloric takes place.”

The amount of heat entering the engine at high temperature is equal to that leaving it at low temperature. This statement is correct (for a reversibly working engine) if we identify Carnot’s heat with entropy. Furthermore, if we identify his *puissance motrice* with the flux of the mechanically released energy *P*, we can write his balance in modern terms:

(12) $$P=\Delta T\cdot I_{S}$$

where $I_{S}$ is the entropy current, and ΔT is the temperature difference between the thermal input and the thermal output of the heat engine.

#### 4.1.3. Joule, Mayer, Helmholtz—Heat Becomes Energy

However, only two decades later, Carnot’s findings were challenged. Originally the cause was a positive incidence. It was realized that what until then had been called work, or force, or puissance motrice, was a manifestation of a state variable, called energy. Several researchers were involved in the development, especially Mayer [16], Helmholtz [17] and Joule [18].

Yet, the new quantity had a characteristic which distinguished it from other physical quantities: It did not seem to measure a certain, well-defined property, but manifested itself sometimes in one way and sometimes in another. It was thus a measure sometimes for one property and sometimes for another one. This apparent peculiarity was the cause of many misunderstandings related to energy [4].

The problem was solved only some decades later, namely when Einstein’s energy–mass equivalence made it clear which properties were actually measured by energy: the same as those measured by mass, namely inertia and gravity (which afterwards, with the General Theory of Relativity, turned out to be one and the same property). Returning to the year 1850: the properties that seemed to be measured by the energy were, from today’s point of view, the properties of other physical quantities: velocity, temperature, and electric voltage, etc., or, to express it with the corresponding extensive quantities: momentum, electric charge, and entropy, etc. Therefore, one could calculate the values of the energy in the most different situations from other quantities, and conclude that for energy a conservation law is valid. One could establish a balance for processes by using a quantity which otherwise had no further property, perhaps comparable with the quantity “monetary value.”

The conservation of the energy was formulated in the form of a theorem called the first law:

(13)  dU= δQ−δW

where δW is the previously known work or force motrice. δQ is a contribution to the energy change caused by the supply of heat. Thus, the new insight was: Q is the quantity of heat that had been searched for for a long time; the nature of heat is energy; and heat is a particular kind of energy.

The fact that neither W nor Q constituted the energy content of a system was, and still is, the cause of many misunderstandings and learning difficulties [19].

Now, the interpretation of δQ  as heat was in contradiction with the old heat concept of Black and Carnot. In retrospect, what happened was this: the name of an existing quantity was taken away from this quantity and given to another one. However, the old quantity was not given a new name, resulting in its disappearance from the scene.

One of the causes of this mishap was that it was not yet known that two extensive quantities are always needed to describe a process, one of which is the energy, an insight that only became clear through Gibbs’ work (see, for example [20,21]).

Gibbs’ fundamental equation is:

(14) dE=TdS−pdV+μdn

or in a form extended to electrical and mechanical processes:

(15) dE=TdS−pdV+μdn+vdp+UdQ

This tells us that every energy exchange is linked to the exchange of at least one more extensive quantity. In the case of a purely thermal process, the relation reduces to:

(16) dE=TdS .

In fact, a similar scenario had already taken place about 150 years before, when Leibniz initiated the famous dispute about the true measure of force. It was about the question: which of the quantities, which today are called momentum and kinetic energy, is the correct measure for the amount of motion [22]. Today we know that we need both, and that they are related in a way analogous to Equation (16):

(17) dE=vdp

where **v** is the velocity and **p** the momentum.

But let us return to the new conviction that the true nature of heat is a form of energy. Heat engines were from now on described by their energy balance. The new energetic heat decreases between the heat input and heat output of the engine. Thus, the beautiful analogy to the water wheel (and to all other energy converters) was no longer recognizable and Carnot was accused of being mistaken. This is still written in the *Lectures on the Theory of Heat* by Helmholtz, published as a book in 1903 [23]:

“According to Carnot’s conception, the body would have received on the path AB an amount of heat *…* from a heat reservoir $R_{1}$ … of temperature $\vartheta_{1}$ as it would have released on the path CD to a heat reservoir $R_{0}$ of temperature $\vartheta_{0}$*…* He concluded that heat could perform external work if it was transferred in unchanged quantity from bodies of higher temperature (the reservoir $R_{1}$) to one ($R_{0}$) of lower temperature, as if it had a state of greater elastic tension at higher temperature and expanded when it passed to lower temperature, whereby the elastic tension of the heat substance is converted into external work. Carnot’s consideration is correct insofar as with this gain in work a certain quantum of heat must necessarily pass from a warmer to a colder body. Yet, it is wrong insofar as the working body mediating the heat transfer does not give off as much heat at the lower temperature $\vartheta_{0}$ as it has absorbed at $\vartheta_{1}$; it gives off less heat, and the difference is equivalent to the external work gained.”

Apparently, another prejudice contributed to the conflict with Carnot’s view. Carnot assumes, it was said, that there is a heat substance, and from a substance one expects its amount to be conserved. Helmholtz, for example, formulates [17]:

“The material theory of heat must necessarily consider the quantity of the heat substance as constant.”

In retrospect, it is clear that the quantity of a substance or the quantity of matter does not have to satisfy a conservation law. This is evident, for instance, in the process of pair production or annihilation. The fact that the caloric is not conserved can therefore not be an argument against Carnot. 

By the way, when dealing with extensive physical quantities, for example mass or electric charge, it is common to use a language with which one normally describes the behavior of substances, yet, neither mass nor electric charge are substances, rather, they are both variables in our theoretical description of the world. Nevertheless, talking about them as if they were substances is of great benefit to our understanding. When doing so, we use a model that we call the *substance model*. In this respect, it is also convenient to talk about heat as if we were talking about a substance. When doing so, we must not forget that the substance model does not require that the imagined heat substance be conserved. 

#### 4.1.4. Clausius—Introduction of the Entropy

It soon became obvious that something had gone wrong: a physical quantity was missing, namely the second extensive quantity for the description of heat exchange processes. It was reintroduced by Clausius under the name of entropy in the years 1854 to 1862, unfortunately in a way that did not reveal its original simplicity [5,24]. It is clear that Black’s and Carnot’s definitions of “heat” satisfy the requirements of our profile. However, at first it was not clear that the properties of the new quantity, entropy, largely coincided with those of Carnot’s and Black’s heat. 

Several decades passed before it was realized that the introduction of the entropy represented a resurrection of Black’s heat or Carnot’s caloric.

### 4.2. Act Two: Resurrection without Consequence

#### 4.2.1. Ostwald: Entropy as the Resurrected Caloric

The first scientist to notice the concordance of Clausius’ entropy and Carnot’s caloric was most likely Ostwald. In his booklet from 1908, *Die Energie* [25], he writes:

“However, the quantity of the thermodynamic theory, which could be compared with the amount of water, is still completely unfamiliar to the general public. It has received the scientific name entropy and plays a role corresponding to its meaning in the theory of thermal phenomena. However, in school and thus in the knowledge of the average-educated, the use of this quantity has not yet gained acceptance and so the information here must be that it really is comparable to the amount of water in so far as its amount does not change as it passes through the (ideal) machine.”

Finally, in 1911, two publications that complemented each other but were independent of each other, brought more clarity to the debate.

#### 4.2.2. Callendar: Entropy for the Schoolboy

Apparently without knowing Ostwald’s book (it was available only in German), H. L. Callendar arrived at the same conclusion [26]: that Clausius’ entropy was the same physical quantity as Carnot’s caloric, and he put forth the remarkable admission that every beginner can gain an understanding of this supposedly difficult quantity:

“Finally, in 1865, when its importance was more fully recognised, Clausius … gave it the name of ‘entropy’, and defined it as the integral of dQ/T. Such a definition appeals to the mathematician only. In justice to Carnot, it should be called caloric, and defined directly by his equation $W=AQ\left(T\;–\;T_{0}\right)$, which any schoolboy could understand. Even the mathematician would gain by thinking of caloric as a fluid, like electricity, capable of being generated by friction or other irreversible processes.”

#### 4.2.3. Jaumann: The Local Balance Equation

Finally, in the same year, Jaumann published an article [27] in which the substance character of entropy was expressed in mathematical form. Shortly thereafter, Lohr [28] established the local balance equation for entropy. Päsler, who extended the “Lectures on Thermodynamics of Max Planck” [29] in the chapter “Remarks on the Thermodynamics of Irreversible Processes”, acknowledged the work of Jaumann and Lohr.

Jaumann’s work corresponded to the questions of that time. In the two decades before, it had been possible to formulate the local balance for energy [30], and three years before, Planck had identified the force as a momentum current [31], so that the old “equations of motion” (Euler, Navier-Stokes) could now be read as local momentum balance equations.

#### 4.2.4. The Preliminary End

The three publications mentioned above had almost no consequences for the teaching of thermodynamics. They fell into oblivion. Callendar himself did not seem to care enough about the subject to work on spreading the idea. Jaumann’s work is not easy to read, but the somewhat clearer work of his student Lohr was not received any more favorably.

Entropy retained its bad reputation as difficult to understand and in a way mysterious. There seemed to be no interest in demystifying it. In the following decades Carnot’s work was analyzed again and again, always with the same result: his caloric can be identified with entropy [7,8,9,10,11,12]. Thereby, Ostwald’s and Callendar’s ideas were sometimes mentioned. In other cases, the later authors seem to have been unaware of their predecessors.

### 4.3. Act Three: Second Resurrection

The next chance for entropy to be considered a measure of heat came in 1972, when Georg Job, a young thermodynamicist from Hamburg noticed that the properties of entropy correspond almost perfectly with what is colloquially called heat, and that it has exactly the properties that a scientist—a physicist or chemist—expects from a measure of the amount of heat. This not only meant that entropy corresponds to Carnot’s caloric, it was also emphasized for the first time that the name heat fits poorly to the process variable Q, whereas it fits perfectly to the state variable *S*.

Fortunately, Job did not write a journal article about his insight; he wrote a textbook [32]. In this book it turned out quite clearly that the generally unloved science of heat becomes surprisingly clear and simple. One realizes that one already has a broad experience from everyday life for dealing with entropy, provided that one is told at the very beginning that entropy measures what is colloquially and intuitively called “amount of heat.” 

It is remarkable that Job, when he wrote his book, did not know the work of Callendar or Jaumann and so we can say that the good idea came up several times but died out again.

While the previous publications were concerned with analyzing and classifying Carnot’s work, Job had a different goal: how to teach thermodynamics while knowing that entropy allows this simple descriptive interpretation. In fact, his book received more attention, and it had the effect of stimulating numerous other authors to address the subject. 

Thus, several articles were written in journals referring to Job’s work [33,34,35,36], several university textbooks were written [37,38,39], and a physics course for high schools was created and tested under government supervision for several years [40].

## 5. Consequences for Teaching

We cannot describe in this essay the details of the physics lessons at school or university, and instead we refer to the given literature.

Nevertheless, we want to make some general remarks regarding the teaching process.

### 5.1. Integration into the Physics Canon

Physics is full of analogies and analogies are important: they simplify the structure of the teaching contents and they simplify the learning process. Analogies in physics usually involve physical quantities corresponding to each other, as well as relationships between these quantities. Let us address an analogy that is essential for embedding thermodynamics into the physics canon (see Table 1).

In the case of the analogy we consider here, firstly the extensive quantities electric charge Q, momentum p, entropy *S* and amount of substance *n*, correspond to each other, (see the second column of Table 1). They have in common that for each of them a local balance can be established, i.e. that for each of them a “continuity equation” holds. This means that to each of them belongs a current, a density and a current density. The respective currents are listed in the third column: the electric current I, the momentum current or force F, the entropy current $I_{S}$ and the matter current $I_{n}$. 

In addition, an “energy conjugate” intensive quantity can be defined for each of the extensive quantities. These are the well-known quantities electric potential ϕ, velocity v, absolute temperature *T* and chemical potential μ (column 4 of Table 1).

Among the numerous relations analogous to each other, let us consider only one, namely that in the fifth column of the table. It describes energy transports: one electrical, one mechanical, one thermal and one chemical. Here P is the energy current. The traditional way to describe these transports is to speak of energy as having different “forms”. The equations suggest a different way of speaking. After all, it is not the energy in which the various transports differ. The difference consists in the fact that the energy flow is accompanied in each case by the flow of another extensive quantity. It is therefore awkward to attribute properties to the energy which are actually properties of the second flowing quantity [4].

Each row of the table characterizes one of the classical sub-domains of physics, including chemistry. 

The table illustrates the role that entropy plays, or could play, in the teaching of physics. It shows that entropy appears as the central quantity of thermodynamics. Let us express it this way:

- Electricity is about electric charge and its currents;
- Mechanics is about momentum and its currents (“forces”);
- Thermodynamics is about entropy and its currents;
- Chemistry is about the amount of substance and its currents, and about reactions.

For the teaching of thermodynamics, which is our actual concern, we give the following recommendations: entropy should be the central quantity of thermodynamics, just as, for example, electric charge is the central quantity of electricity. To state this more emphatically: thermodynamics without entropy and without entropy currents is like electricity without electric charge and without electric currents. One may object that electricity without electric currents is senseless or impossible. Fuchs [33] shows in an amusing paper that it is indeed possible. Of course, his concern is not to present a new theory of electricity, but to reveal the lamentable condition of thermodynamics.

### 5.2. Surrogate Quantities and Substitute Constructions

Since entropy is widely believed to be an obscure quantity, one tries to get along with substitute or surrogate constructions for processes which could be better described with entropy. We have already mentioned enthalpy and thermal energy, but the heat Q can also be identified as such a substitute. In the following, we give some more examples of how one tries to avoid using the entropy (see also [41]). 

#### 5.2.1. Energy Dissipation

A process is known to be irreversible when entropy is produced. This is easy to understand: entropy is the only physical quantity we know of that defines a direction in time; it can be created, but it cannot be annihilated. All other extensive quantities can either be neither produced nor annihilated (such as electric charge, momentum and energy), or they can be both produced and annihilated (such as the amount of substance).

Instead of stating this simple fact to make the entropy responsible for the irreversibility, one shifts the cause to the energy and claims that energy is dissipated. It is true that dissipation is a process which runs spontaneously and does not run backwards, yet exactly this effect can be described by the production of entropy. 

#### 5.2.2. Energy Loss

If entropy is unintentionally generated in a stationary process, for example in a frictional process, it must be carried away and deposited somewhere in the environment. The corresponding entropy flow is inevitably associated with an energy flow, and the energy flowing away in this manner is what is known to be the energy loss of the process under consideration. It is calculated as:

(18) $$P_{L}=T_{0}I_{S}$$

$T_{0}$ is the temperature of the environment, i.e., the temperature of the system into which the entropy is discharged. Usually, one is interested in the loss in relation to the incoming energy flow. Thus, one can define a dimensionless energy loss *L* whose value is between 0 and 1:

(19) $$L=\frac{T_{0}I_{S}}{P}$$

where P is the inflowing energy current.

Instead of the energy loss, one can also describe the situation by an “efficiency”:

(20) $$\eta =1-\frac{T_{0}I_{S}}{P}$$

Unfortunately, this is referred to by the somehow intimidating name of “second law efficiency”, and comes up in the lecture, if at all, in a rather advanced context. 

Much more popular in thermodynamics is another “efficiency”, the Carnot efficiency:

(21) $$\eta_{C}=\frac{T_{2}-T_{1}}{T_{2}}\;.$$

It is usually explained with the help of the reversibly working Carnot engine.

It seems to state that a heat engine has in principle an efficiency lower than 1. It cannot be increased by improving the engine; although the engine has no defect, the efficiency is smaller than 1.

This incongruity is resolved if we look not at the Carnot engine alone, but at the entire installation, consisting of the Carnot engine and a furnace. Let us calculate the efficiency of the combustion process alone, using Equation (20). Since all the energy available by the combustion process is used for the production of entropy, we have:

(22) $$P=T_{2}I_{S}$$

According to Equation (20), and renaming the temperature of the environment $T_{1}$ instead of $T_{0}$ we get:

(23) $$\eta =1-\frac{T_{1}I_{S}}{T_{2}I_{S}}=\frac{T_{2}-T_{1}}{T_{2}}=\eta_{C}$$

Thus, it is not the Carnot engine that is to blame for the poor efficiency, but the combustion process in the furnace. It is the combustion where the sin is committed, where entropy is produced. The so-called Carnot efficiency (which, by the way, was not introduced by Carnot) is not the efficiency of the Carnot engine but that of the combustion process. 

This suggests to replace the recommendation of “save energy” with “avoid entropy production”.

In fact, the whole system, which is supplied with fuel and oxygen, can in principle be operated reversibly, i.e., without entropy production: one does not burn the fuel in a freely running reaction, but feeds it into a fuel cell (which we may imagine as working reversibly). Then, the efficiency of the system becomes equal to 1.

#### 5.2.3. The Principle of Minimum Energy

Finally, there is another habit which indicates that one does not like to use the entropy balance as an argument. Whenever an equilibrium is establishing, entropy is being produced; as much as is possible under the given boundary conditions. However, instead of saying that a system evolves toward the state of maximum entropy, the process is usually described by saying that it runs into a state of minimum energy.

Moreover, the formulation with the energy minimum must contain a catch. If during a process of the establishment of an equilibrium a system passes into a state of minimum energy, then, because of energy conservation, another complementary system, usually the environment, must inevitably pass into a state of maximum energy. One might ask why the minimum energy theorem is valid for one system and its opposite for the other. 

### 5.3. University Students’ Problems with Thermodynamics

We conducted a short survey in a group of 15 students. Every student was to rate for different subfields of physics, first how interesting the student finds the field, and second how competent the student feels in that subfield. (We compared only the scores for mechanics, thermodynamics and modern physics.) The result was as we had suspected (see Table 2).

Thermodynamics is perceived as no less interesting than mechanics, but students feel much less competent. One can see that something must be wrong with our teaching. 

We also found in numerous discussions with students that they were not able to explain why it is meaningless to speak of the heat content of a physical system. Also, they were not aware of the fact that entropy is a quantity for which a balance can be established. In other words, they did not know any thermodynamic quantity with this property.

### 5.4. Teaching Practice

Although thermodynamics courses introducing entropy as a measure of heat are now given at various universities [37,38,39], we only report about our experience in high school teaching, since here the number of learners was much larger, and thus much more experience is available [35,40].

Another advantage of testing a teaching concept at high school is that one gets more direct feedback from the learners. At school, one simply cannot afford to lecture in an incomprehensible technical jargon, as is quite possible at the university. Incidentally, the difficulties that university students have with thermodynamics are the same as those that one encounters with high school students.

At the high school, we introduce the entropy together with temperature in the very first lesson of thermodynamics. It is explained that in thermodynamics, as in the other areas of physics, a measure of a quantity of something is needed; in the case of thermodynamics a measure for what is contained in a body due to its hotness. One could call it “quantity of heat”; however, in physics, one likes to use special designations for the sake of unambiguity. Therefore, this quantity of heat is called entropy. 

Next, properties of the entropy and its behavior in various processes are studied and discussed, always guided by the ideas about the notion of heat in our everyday experience. One finds:

- Among two bodies of the same temperature, differing only in size, the larger one contains more entropy;
- Entropy flows spontaneously from warm to cold. If one wants it to flow from cold to warm, one needs a heat pump;
- Entropy can be generated in many different ways, but it cannot be annihilated. When teaching, one realizes that this statement, which seems so transcendent to the physicist, can be concluded solely from the everyday experience of the students;
- With the help of a cryogenic machine (a heat pump) one can extract entropy from a body, whereby its temperature decreases. However, one does not succeed in achieving a temperature below −273 °C. The reason for this is that the body contains no more entropy. We introduce the absolute temperature scale;
- Entropy transport by conduction and by convection is discussed.

All of this covers about eight lessons. Notice that the second and the third laws have been introduced in these lessons. Thereafter, we discuss the relation between entropy and energy, expressed in the equation:

(24) $$P=T\cdot I_{S}\;.$$

This can be used to understand heat engines and heat pumps, and their energy and entropy balances.

Thereafter, the students learn how to measure entropy differences by applying Equation (24), for example the difference of the entropy content in a bucket of water at 20 °C and at 80 °C. To do this, they need equipment, which can be found in any home: an immersion heater, a thermometer, and a stopwatch. In principle, however, the same procedure is also used to determine the absolute values of the entropy. 

We do not introduce the quantity Q, that is the process quantity traditionally called heat, nor do we introduce the quantity work. Both have always been alien bodies in physics, and they are not needed in a modern curriculum.

In retrospect, one realizes that entropy is not a difficult physical quantity, as it is reputed to be; it is one of the easiest, most descriptive quantities in physics, provided it is introduced appropriately.

## 6. Conclusions

Entropy is the quantity that best satisfies the expectations that we have for a measure of the amount of heat. All the competing quantities have shortcomings, some of them considerable ones. This is especially true for the quantity Q. Using the term “heat” for Q raises expectations that this quantity cannot fulfill.

A didactic approach at high school and university, where entropy is introduced as a measure of heat right at the beginning of thermodynamics, considerably gains in simplicity and comprehensibility. It is not necessary to rename the quantity. It is sufficient to emphasize from the outset that entropy largely corresponds to what is colloquially understood by heat.

Let us conclude with the words of Ostwald:

“In any case, it is a hard work to develop a new scientific view in a more or less known area and to consider the old phenomena in a new light; it is as if one had to describe a familiar content in a new language, which one first has to learn for this purpose. What a wonder, if one tries again and again to keep the way of expression of the old language and to apply the new one only where it cannot be otherwise, because the old one had been inadequate.”

## Figures and Tables

**Table 1:** Analogy between four subfields of science.

	Extensive Quantity	Current	Intensive Quantity	Energy Current
electricity	Q	I	ϕ	P=U · I
mechanics	p	F	v	P=v · F
thermodynamics	S	$I_{S}$	T	$P=T\;\cdot \;I_{S}$
chemistry	n	$I_{n}$	μ	$P=\mu \;\cdot \;I_{n}$

**Table 2:** Results of a survey with University students.

	Find it Interesting	Feel Competent
mechanics	4/10	9/10
thermodynamics	4/10	2/10
modern physics	9/10	3/10

## Data Availability

Not applicable.
